# Outpatients' satisfaction with healthcare services received at a district hospital in Botswana

**DOI:** 10.4314/gmj.v56i3.12

**Published:** 2022-09

**Authors:** Zibo K Khuwa, Sogo F Matlala, Thembelihle S Ntuli

**Affiliations:** 1 Department of Public Health, University of Limpopo, Private Bag X1106, Sovenga, 0727, South Africa; 2 Department of Statistical Sciences, Sefako Makgatho Health Sciences University, P.O Box 60, Medunsa, 0204, South Africa

**Keywords:** District hospital, healthcare professionals, patient satisfaction, quality healthcare services

## Abstract

**Objectives:**

To investigate patient satisfaction regarding healthcare services at a district hospital. The research question was: what is the level of patient satisfaction regarding service delivery?

**Design:**

An observational cross-sectional descriptive study conducted in September 2019.

**Settings:**

A district hospital in Botswana serving a population of 90 000. Outpatients from the Eye clinic, Casualty and Outpatient Department, Sexual Reproductive Health clinic and Infectious Diseases Control Centre were selected for the study

**Participants:**

240 stable outpatients over 17 years selected through consecutive sampling participated voluntarily after giving informed consent.

**Main outcome measures:**

The level of satisfaction was measured using 19 questions on five-point Likert scales ranging from strongly disagree 1, disagree 2, unsure 3, agree 4 to strongly agree 5. A binary outcome was created into satisfied and unsatisfied using the mean score as the cut-off point. Age, gender, employment, education and departments were independent variables.

**Results:**

65% (95% CI: 58–71%) were satisfied but unsatisfied with: doctor's politeness (66.9%; 95% CI: 60–73%), explaining (67.8%; 95% CI: 61–73%), privacy (65.6%; 95% CI: 59–72%), skills (67.4%; 95% CI: 61–73%), confidence (67.4% 95% CI: 61–73%), compassion (66.5%; 95% CI: 60–72%) and waiting time (49.2%; 95% CI: 42–57%). Department visited predicted satisfaction (p=0.002); those from the Eye clinic and Sexual Reproductive Health clinic were satisfied compared to others.

**Conclusion:**

Satisfaction was generally high but lower regarding specified services and departments visited. There is a need for targeted interventions. Studies are needed to explore reasons for lower satisfaction in Casualty, Outpatient Department and Infectious Diseases Control Centre.

**Funding:**

None declared

## Introduction

Patients deserve and expect quality healthcare services from hospitals. Thus, they become dissatisfied if they receive poor-quality healthcare services at the hospitals they visit. Patient satisfaction is one of the instruments used to measure the expectations and experiences of patients on the quality of healthcare services they receive, and it can help to improve the quality of healthcare services delivered.^1^–^3^ This suggests a relationship between patient satisfaction and the quality of healthcare services received.

Studies from low- and middle-income countries report various patient satisfaction rates of below 60%,3,^4^ rates between 60% and 80%,^2^,^5^ while other studies found satisfaction rates of above 80%.^6^–^8^ A 2011 study in a Botswana primary health facility found that most participants were satisfied (mean score = 3.75) with the services provided by the different service providers, even though different methods were used to measure patient satisfaction.^9^ In high-income countries, patient satisfaction rates of 69.3% and 96.3% were reported in Australia and China, respectively.^10^,^11^

Lack of economic growth, shortage of medical products and technologies, and a shortage of competent healthcare workers are some factors that influence the delivery of quality healthcare services in low-and middle-income countries.^12^,^13^ Botswana is not an exception. The country is experiencing a shortage of experienced nurses and doctors due to, among others, failure to attract, retain and properly use healthcare workers and poor working conditions and a shortage of medical products and technologies in some healthcare institutions.^14^,^15^ This shortage affects the delivery of quality healthcare services; hence this study aimed to investigate the level of patient satisfaction regarding healthcare services received at a district hospital in Botswana, which had a shortage of nurses and doctors.

## Methods

### Study design and Study setting

This facility-based cross-sectional study was conducted at a district hospital in Botswana for one month in September 2019. The district hospital had 50 beds and included a Paediatric ward, a Maternity ward, and Casualty and Outpatient Department. Other departments included Sexual Reproductive Health, Infectious Diseases Control Centre; Eye clinic; Dental clinic and Operating Theatre. The hospital was staffed by 73 nurses and 9 doctors serving over 90 000 population in the catchment area. Patients pay a minimal fee of 10 Botswana pula (0, 85 USD) if they can afford it; otherwise, they receive free healthcare services as healthcare is subsidised.

### Study population, inclusion, and exclusion criteria

The study populations were outpatients who received healthcare services at the district hospital during the study period. Patients under 18 years and unstable psychiatric patients were excluded from participating in the study because they could not give informed consent.

### Sample size and sampling technique

The minimum sample size for the study was 196, calculated using the formula: n = Z^2^ p (1-p)/e^2^. The sample size was determined by considering the patient satisfaction rate of 86.6% reported in other studies,^2^, ^3^,^7^, ^8^ 95% confidence interval and a 5% margin of error. A non-response rate of 10% was expected. The calculated sample was allocated proportionally to the departments. A consecutive sampling technique was used to administer the questionnaire to patients who received treatment and completed their outpatient visits at Eye Clinic, Infectious Diseases Control Centre, Casualty and Outpatient Department, and Sexual Reproductive Health. These four departments were selected as they were places where doctors and nurses provided healthcare services.

### Data Collection

A self-administered questionnaire developed based on literature review 2,11 was used to collect data. The questionnaire comprised socio-demographics and 19 items to assess the level of satisfaction measured on a five-point Likert scale ranging from strongly disagree 1 disagree 2, unsure 3, agree 4 to strongly agree 5. The questionnaire was prepared in English and then translated into Setswana, the local language. It had a Cronbach alpha of 0.90.

### Data Analysis

Data were entered and analysed using Microsoft Excel Spreadsheet and SPSS version 21, respectively. The total score of all 19 items was dichotomised into satisfied and unsatisfied using the mean score as a cut-off point, so a score equal to or above the mean value was considered satisfied or otherwise unsatisfied. A comparison between groups was performed using Chi-square/Fisher exact tests, and a p-value of less than 0.05 was considered statistically significant.

### Ethical Clearance

This study was part of the first author's Master of Public Health (MPH) research project. As such, ethical approval (TREC/145/2020: P.G.) was obtained from the Turfloop Research Ethics Committee of the University of Limpopo where the degree was offered. Permission to conduct the study was sought from the Botswana Ministry of Health (HPDME 13/18/1VI 25) and hospital management (PDHMT PF/42 1 59) before the beginning of data collection. Informed consent was obtained from all participants. Participants were given information about the study in English and Setswana to obtain informed consent. Participants were protected from psychological and physical harm, including protection from infection with coronavirus.

As data were collected during the SARS-CoV-2 pandemic, measures to prevent the transmission of COVID-19 were taken. Both participants and the researcher put on face masks as putting on a mask to cover the nose and the mouth is a requirement in Botswana when entering public areas.^16^ The hands of the researcher and participants were sanitised, while the pen used was wiped with an alcohol-impregnated wipe after each use.

## Results

### Respondent characteristics

Two hundred and forty (240) outpatients participated in the study (Table 1). Their mean age was 38.5±15.6 years ranging from 18 to 87 years. More than half (59%) were less than 40 years old, 62% were females, and 38% were males. Nearly half (47%) were employed, 39% were unemployed, and 14% were students. Forty-two per cent had secondary school education, and nearly forty per cent (38%) had tertiary education. The Casualty and Outpatient departments accounted for 33% of the participants.

**Table 1:** Demographic characteristics of patients, n=240

Variable	Frequency (Percentage) n (%)
**Age (years)**	
<20	17(7)
20–29	68(29)
30–39	56(23)
40–49	45(19)
50–59	27(11)
60+	27(11)
**Gender**	
Male	92(38)
Female	148(62)
**Employment Status**	
Unemployed	93(39)
Employed	112(47)
Student	35(14)
**Level of Education**	
None	32(13)
Primary	17(7)
Secondary	99(42)
Tertiary	92(38)
**Departments**	
Eye clinic	50(21)
Infectious Diseases Control Centre	60(25)
Casualty and Outpatient Department	80(33)
Sexual Reproductive Health	50(21)

### Level of satisfaction with health service delivery

As Table 2 shows, respondents strongly disagree and disagree with the following items: the doctor who treated me was polite (66.9%), the doctor explained to me what was wrong with me (67.8%), and the doctor respected my privacy (65.6%), I'm satisfied with doctor's expertise/skill (67.4%), I have confidence on the doctors (67.4%), the doctor who attended me showed a sense of concern (66.5%) and (49.2%) the waiting time to be assisted was reasonable.

**Table 2:** Individual items of outpatient satisfaction with service delivery

	N	Mean±SD	Strongly Disagree	Disagree	Unsure	Agree
The casualty and outpatient department has convenient operating hours	237	3.58±0.61	4(1.7)	3(1.3)	80(33.8)	150(63.3)
The doctor who treated me was polite	239	2.48±0.76	1(0.4)	160(66.9)	40(16.7)	38(15.9)
The nurse who treated me was polite	239	3.58±0.64	4(1.7)	7(2.9)	74(31.0)	154(64.4)
I am pleased with the way I was treated at the hospital	236	3.59±0.63	6(2.5)	-	77(32.6)	153(64.8)
The doctor explained to me what was wrong with me	239	2.47±0.75	1(0.4)	162(67.8)	39(16.3)	37(15.5)
The nurse explained to me what was wrong with me	240	3.54±0.62	3(1.3)	7(2.9)	87(36.3)	143(59.6)
The doctor respected my privacy	240	2.50±0.79	2(0.8)	158(65.8)	38(15.8)	42(17.5)
The nurse respected my privacy	239	3.61±0.58	1(0.4)	9(3.8)	71(29.7)	158(66.1)
Next time I am ill I will come back here	240	3.58±0.61	3(1.3)	6(2.5)	81(33.8)	150(62.5)
I am satisfied with doctor's expertise/skill	239	2.45±0.74	2(0.8)	161(67.4)	42(17.6)	34(14.2)
I am satisfied with nurse's expertise/ skill	238	3.56±0.58	1(0.4)	8(3.4)	86(36.1)	143(60.1)
Waiting time to be assisted is reasonable	179	2.27±1.25	82(45.8)	6(3.4)	51(28.5)	40(22.3)
I am satisfied with the services provided	237	3.50±0.69	8(3.4)	4(1.7)	86(36.3)	139(58.2)
I have confidence on the doctors	239	2.46±0.75	2(0.8)	161(67.4)	40(16.7)	36(15.1)
I have confidence on the nurses	239	2.59±0.61	3(1.3)	7(2.9)	76(31.8)	153(64.0)
Time taken for consultation was good	239	3.52±0.76	12(5.0)	3(1.3)	72(30.1)	152(63.6)
Explanation about my condition was good	239	3.61±0.58	3(1.3)	2(0.8)	80(33.5)	154(64.4)
The doctor who attended me showed sense of concern	239	2.46±0.74	2(0.8)	159(66.5)	45(18.8)	33(13.8)
The nurse who attended me showed sense of concern	238	3.52±0.63	3(1.3)	8(3.4)	89(37.4)	138(58.0)

### Socio-demographic factors influencing the level of satisfaction

After dichotomising the total score of the 19 items of satisfaction into satisfied (mean score ≥58.9) and unsatisfied (mean score <58.9), nearly two-thirds (65%) of the respondents were satisfied with the health care service they received and only 35% were unsatisfied. As shown in Table 3, there was no statistically significant difference between age, gender, employment status and level of education with the level of satisfaction. A significantly high proportion of respondents seen in the eye clinic were more satisfied compared with those in other departments (p<0.05).

**Table 3:** Level of patient satisfaction with healthcare services

	Outpatient level of satisfaction		p-value
Variable	Satisfied n(%)	Unsatisfied n(%)	
**Age (years)**			0.446*
<20	14(82)	3(18)	
20–29	43(63)	25(37)	
30–39	37(66)	19(34)	
40–49	28(62)	17(38)	
50–59	14(52)	13(48)	
60+	19(70)	8(30)	
**Gender**			
Male	60(65)	32(35)	0.871
Female	95(64)	53(36)	
**Employment Status**			0.517
Unemployed	56(60)	37(40)	
Employed	76(67)	36(32)	
Student	23(66)	12(34)	
**Level of Education**			0.274
None	21(66)	11(34)	
Primary	7(41)	10(59)	
Secondary	64(65)	35(35)	
Tertiary	63(68)	29(32)	
**Departments**			
Eye clinic	42(84)	8(16)	0.002
Infectious Diseases Control Centre	33(55)	27(45)	
Casualty and Outpatient Department	44(55)	36(45)	
Sexual Reproductive Health	36(72)	14(28)	

* Fisher Exact

## Discussion

The main purpose of this study was to determine patients' satisfaction levels using a district hospital in Botswana. The mean age of the participants was 38.5 years (SD: 15.6) which ranged from 18 to 87 years. A similar study conducted in South Africa reported a mean age of 35.6 years (SD:12.2) ranging from 18 to 75 years,^2^ while in some Ghanian studies, participants had a mean age of 61.2 years (SD:14.5)^17^ and 47 years (SD: 19.5),^18^ respectively. Consistent with previous studies, a greater proportion of outpatients in the present study fell within the age groups <49 years, 2,19 The National Demographic Survey of 2017 carried out in Botswana found that slightly more than half (59.8%) of the population were in the age group 15–64 years.^20^ In contrast, in a Ghanaian study, slightly more than two-thirds (69%) of the participants were in the age group 45 years and older.^17^ The reasons for young adults using this healthcare facility are unclear and need further investigation. This might assist in understanding the burden of diseases in this age group.

In agreement with previous studies,^2^,^17^–^19^,^21^ a greater proportion of the participants in the present study were females. The reason for these could be that, unlike men, women are health conscious, or it could be that women use maternal healthcare services such as cervical cancer screening, antenatal care, postnatal care and family planning.^22^

In this study, more than one-third (39%) of the outpatients were unemployed. The finding of this study is lower than the 54.8% reported in a study conducted in a district hospital in South Africa^2^. In contrast, in Nigeria, a cross-sectional study found that slightly more than half (53%) of the participants were farmers, 19 while in Ghana, 98% of the respondents were employed.^17^

The findings of this study were higher than a household survey conducted in Botswana, which indicated that less than half (40.7%) of the population was unemployed.^20^ Consistent with the finding of a previous study, a higher proportion of the participants had secondary and tertiary education.^2^, ^17^–^18^ In contrast, a Nigerian study found that nearly half (45%) of the participants were illiterates as they could neither read nor write.^19^ Surprisingly, the World Bank^23^ shows that most (75%) of the population in Botswana achieved lower than tertiary education level. Nearly two-thirds (65%) of the outpatients were satisfied with the healthcare services they received in this study.

This finding is comparable to 61% reported in South Africa^2^ but higher than 44% found in Ghana^3^ and 59.3% in Nigeria^4^. Our finding is lower than the satisfaction rate (81%) reported in Western Cape Province, South Africa7, 86.6% in India8, 90% in Ghana17 and rates between 70% and 80% in Ethiopia^6^. Another Nigerian study that assessed patient waiting and service times in the outpatient general Ophthalmology clinic of the University of Ilorin teaching hospital found that more than half (53%) of the patients were satisfied with the total time spent at the clinic^18^. The differences in patient satisfaction levels in these studies might be due to differences in study settings, definitions used for patient satisfaction and sample sizes.

Concerning the individual items of health care received, in South Africa, a cross-sectional study conducted in a district hospital indicated that slightly more than half (51.7%) of the outpatients said that all the staff respected their privacy, and 89.2% said that the doctor who treated them was polite. In 85.2% the doctor explained to them what was wrong with them^2^. In an earlier Botswana study, 38% of the patients were satisfied, while 62% were dissatisfied with the service provided by doctors^9^. In this study, two-thirds (67%) of the participants said that the doctor who treated them was not polite, 66% did not respect their privacy, 68% did not explain to them what was wrong with them, 67% did not show them any sense of concern and 67% of participants said that they did not have confidence on the doctors. The reasons for this attitude to doctors in the present study are unclear and need further investigation.

A cross-sectional study carried out at an oral health centre at tertiary teaching in South Africa found that a long waiting time before consultation with a dentist was associated with decreased patient satisfaction^24^. Xie and Or 25 found in their study at a teaching hospital in China that 89.4% of the time patients spent in the hospital visit was spent waiting to receive healthcare services. Similarly, a study in Fiji also reported that 70% of the patients had to wait more than an hour before being seen by a doctor^11^. In the current study, the long waiting time to be assisted was one of the healthcare services concerns mentioned by nearly half (49.2%) of participants.

Several studies have shown that the rating of the healthcare services received by patients was influenced by age^26^,^19^, gender^27^,^28^, level of education and employment status.^19^ In contrast, other studies showed no significant changes between gender^19^,^21^,^26^, employment status^21^,^26^, level of education^26^ and patient satisfaction. In the current study, age, gender, employment status and level of education were not significantly associated with patient satisfaction. Different specialities/disciplines can influence patient satisfaction concerning the healthcare service received. In this study, a significantly greater proportion of participants seen in the IDCC and OPD were more unsatisfied with the healthcare services received than those in other departments. This finding was supported by a study which showed that patients with psychiatric, hepato-pancreato-biliary and cardiovascular diseases were likely to report poor satisfaction.^29^

This study has several limitations. Firstly, it was carried out in one district hospital, which might limit the generalizability of the findings to other outpatients' healthcare facilities in Botswana. Secondly, since this is a cross-sectional study, it was difficult to determine the causality inferences. Thirdly, the study used consecutive sampling, potentially introducing sampling bias as it is a non-probability sampling technique. Lastly, doctors and nurses might have improved their care practices during data collection, inflating the study's findings.

## Conclusion

The findings of this study indicated that patient satisfaction with the healthcare services received in this healthcare facility was favourably high. However, there is a need for developing interventions in terms of the following items: patient waiting time before receiving services, healthcare workers, particularly doctors' interpersonal competencies, communication skills and patient-centred care.
